# Whole-transcriptome RNA sequencing reveals the global molecular responses and ceRNA regulatory network of mRNAs, lncRNAs, miRNAs and circRNAs in response to copper toxicity in Ziyang Xiangcheng (*Citrus junos* Sieb. Ex Tanaka)

**DOI:** 10.1186/s12870-019-2087-1

**Published:** 2019-11-21

**Authors:** Xing-Zheng Fu, Xiao-Yong Zhang, Jie-Ya Qiu, Xue Zhou, Meng Yuan, Yi-Zhong He, Chang-Pin Chun, Li Cao, Li-Li Ling, Liang-Zhi Peng

**Affiliations:** 1 Citrus Research Institute, Southwest University, Chongqing, 400712 China; 2 Citrus Research Institute, Chinese Academy of Agricultural Sciences, Chongqing, 400712 China

**Keywords:** Citrus, Copper, CeRNA, Transcriptome, Non-coding RNA

## Abstract

**Background:**

Copper (Cu) toxicity has become a potential threat for citrus production, but little is known about related mechanisms. This study aims to uncover the global landscape of mRNAs, long non-coding RNAs (lncRNAs), circular RNAs (circRNAs) and microRNAs (miRNAs) in response to Cu toxicity so as to construct a regulatory network of competing endogenous RNAs (ceRNAs) and to provide valuable knowledge pertinent to Cu response in citrus.

**Results:**

Tolerance of four commonly used rootstocks to Cu toxicity was evaluated, and ‘Ziyang Xiangcheng’ *(Citrus junos*) was found to be the most tolerant genotype. Then the roots and leaves sampled from ‘Ziyang Xiangcheng’ with or without Cu treatment were used for whole-transcriptome sequencing. In total, 5734 and 222 mRNAs, 164 and 5 lncRNAs, 45 and 17 circRNAs, and 147 and 130 miRNAs were identified to be differentially expressed (DE) in Cu-treated roots and leaves, respectively, in comparison with the control. Gene ontology enrichment analysis showed that most of the DEmRNAs and targets of DElncRNAs and DEmiRNAs were annotated to the categories of ‘oxidation-reduction’, ‘phosphorylation’, ‘membrane’, and ‘ion binding’. The ceRNA network was then constructed with the predicted pairs of DEmRNAs-DEmiRNAs and DElncRNAs-DEmiRNAs, which further revealed regulatory roles of these DERNAs in Cu toxicity.

**Conclusions:**

A large number of mRNAs, lncRNAs, circRNAs, and miRNAs in ‘Ziyang Xiangcheng’ were altered in response to Cu toxicity, which may play crucial roles in mitigation of Cu toxicity through the ceRNA regulatory network in this Cu-tolerant rootstock.

## Background

Copper (Cu) is an essential micronutrient for plant growth and development. As a redox-active transition element, Cu plays key roles in photosynthesis, respiration, C and N metabolism, oxidative stress protection, lignification, pollen fertility, and ethylene perception [1–4]. Most of the functions rendered by Cu are ascribed to enzymatically bound Cu, which catalyzes redox reactions [1]. In plants, there are more than 100 Cu-containing proteins, such as plastocyanin (PC), copper/zinc superoxide dismutase (CSD), cytochrome c oxidase (COX), laccase (LAC), diamine oxidases (DAO), and polyphenol oxidases [1–3]. Despite being essential, Cu is easily toxic to plants, even at a supra-optimal level [5, 6]. Excessive Cu inhibits plant growth and uptake of other mineral nutrients and alters enzyme systems, membrane integrity and many other biochemical and physiological processes [5]. Unfortunately, in the last decades, Cu contamination has become a global issue due to the long-term use of Cu-containing fungicides and bactericides, wastewater irrigation, and unconscionable Cu mining [6, 7]. In China, Cu is ranked as the fourth most contaminating heavy metal of agricultural lands [7, 8]. Thus, it is important and pressing to gain better understanding of physiological and molecular responses to Cu excess.

It is worth mentioning that plants have evolutionarily developed a tightly regulated system to balance the uptake, efflux, chelation, distribution, and utilization of Cu [9, 10]. In this system, a number of functional proteins (such as Cu transporters and chaperones), transcription factors (TFs), as well as non-coding RNAs (ncRNAs) may be involved in regulation of Cu homeostasis. For instance, Cu transporters, including CTR-like Cu transporters (COPTs), P-type heavy metal ATPases (HMAs), yellow stripe-like (YSL) proteins, zinc/iron-regulated transporter (ZRT/IRT)-related proteins (ZIPs), and cation diffusion facilitators (CDFs), were shown to directly participate in Cu uptake, transport, and distribution [2, 3, 9, 11]. Among them, COPTs (such as COPT1, COPT2, and COPT6) are mainly responsible for Cu uptake from soil and redistribution to reproductive organs [12, 13]. The HMAs such as AtHMA5, AtHMA7/RAN1, AtHMA6/PAA1, and AtHMA8/PAA2 of *Arabidopsis thaliana* are involved in Cu transport into the xylem, chloroplast, or thylakoid [7, 14–16]. YSL16 protein functions in recycling of Cu from older tissues to young tissues and grains [17]. Apart from these functional proteins, a conserved transcription factor called SPL7 (Squamosa Promoter binding-Like 7) has been shown to be a central regulator of Cu homeostasis [18, 19]. SPL7 regulates the expression of multiple targets, such as COPT1, COPT2, COPT6, cupric reductases FRO4 and FRO5, and Cu-regulated microRNAs (miR397, miR398, miR408 and miR857), that contain reiterative Cu-response elements (CuREs) with a GTAC motif in their promoters under Cu deficiency or excess [18, 20–22].

In addition to the protein-coding RNAs, emerging evidence has revealed that ncRNAs also play essential regulatory roles in plant responses to abiotic stress [23]. MicroRNAs (miRNAs), a major class of ncRNA with a length of 19 to 24 nucleotides, participate in Cu homeostasis by repressing translation or directly degrading Cu-related proteins in plants [22, 24]. Previous studies indicate that Cu deficiency induced the expression of miR397, miR408, and miR857, which repressed a number of Cu-containing proteins, including CSD1, CSD2, LAC, COX subunit 5b (COX5b-1), and Cu chaperone for SOD (CCS1) [20, 25, 26], while Cu excess downregulated miR398 to induce the expression of CSD1 and CSD2 [27]. Recently, two types of ncRNA, long non-coding RNAs (lncRNAs) and circular RNAs (circRNAs), were discovered in plants. LncRNAs belong to a group of ncRNA longer than 200 nucleotides and can regulate the expression of genes through *cis*−/*trans*-acting or miRNA sponges [28, 29]. CircRNAs are endogenous covalently closed circular RNAs that are generated by alternative circularization [30]. A competing endogenous RNA (ceRNA) hypothesis has been proposed that the lncRNAs, circRNAs, mRNAs and pseudogenes can act as ceRNAs to competitively bind common miRNA response elements (MREs), and thus regulate a wide range of biological and developmental processes [31–37]. These ceRNAs are also named miRNA sponges to sequester specific miRNAs and inhibit their functions [30, 31, 34, 35]. In several studies, the ceRNA regulatory theory has been used to uncover molecular mechanisms of plant biology. For example, a ceRNA network was constructed to dissect function of lncRNAs in phosphate starvation of rice [32]. A complex ceRNA network consisting of lncRNAs, mRNAs, and miRNAs was established for maize seed development [33]. A large number of circRNAs, possibly acting as ceRNAs, were found in the ethylene pathway of tomato [38]. Recently, the ceRNA networks were also reported to be related to *A. thaliana* leaf development, tomato flowering, and cucumber heat stress response [35, 37, 39, 40]. However, whether lncRNAs and circRNAs participate in Cu homeostasis by acting as ceRNAs in plants remains to be investigated.

Citrus is widely grown worldwide. With extensive application of Cu-containing bactericides for controlling citrus canker disease, Cu toxicity has become a potential threat for citrus. However, relevant researches to understand Cu toxicity response are greatly limited. In this study, whole-transcriptome RNA sequencing (RNAseq) of Ziyang Xiangcheng (*Citrus junos* Sieb. ex Tanaka), a widely used citrus rootstock in China, was performed so as to uncover the global molecular responses to Cu toxicity at both protein-coding RNAs (mRNAs) and ncRNAs (lncRNAs, miRNAs, and circRNAs) levels. Moreover, the ceRNA networks were constructed for further revealing the underlying regulatory mechanisms in response to Cu toxicity.

## Results

### Evaluation of tolerance to Cu toxicity in the citrus rootstocks

**Fig. 1:**
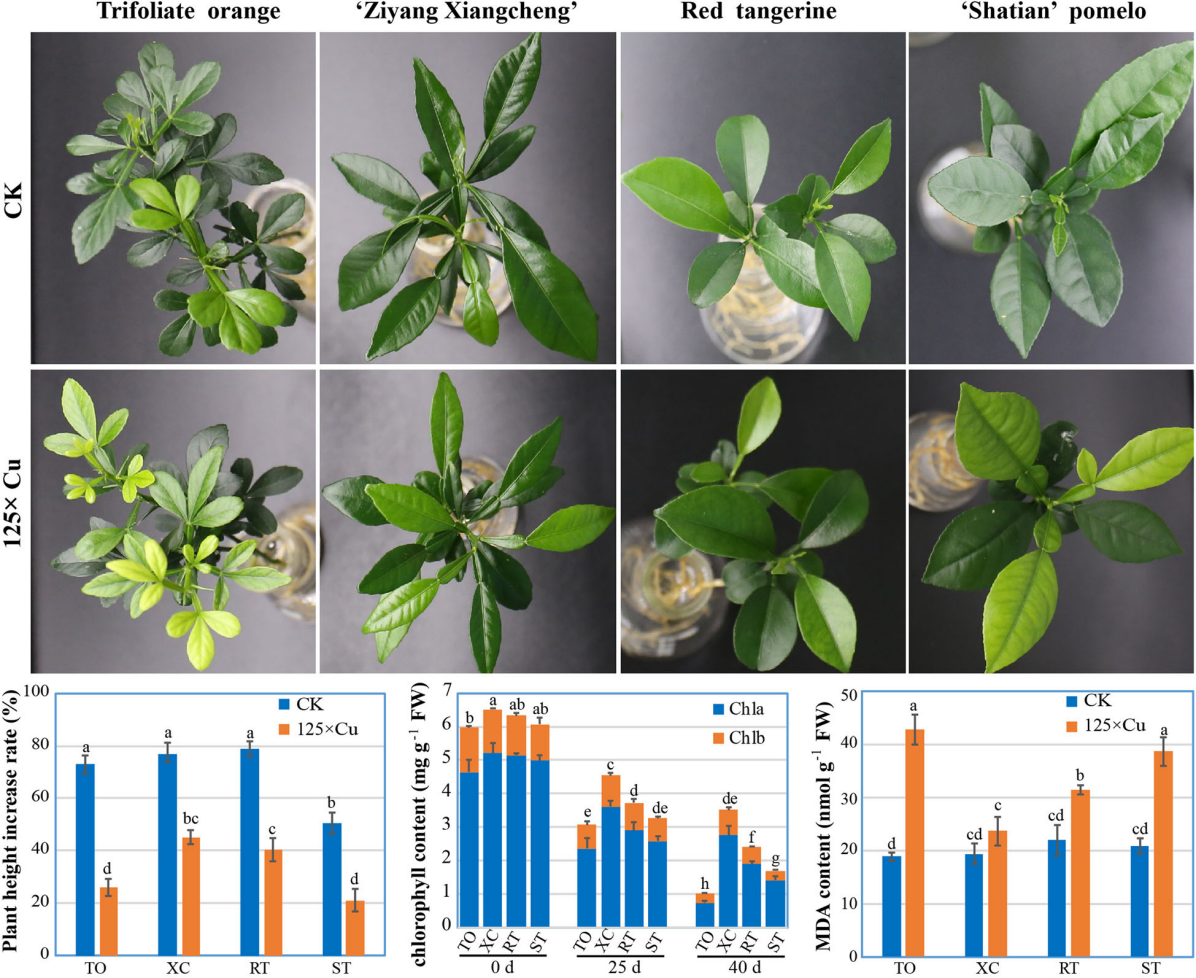
Phenotype and physiological changes of four rootstocks under normal (CK) and Cu toxicity. Four common citrus rootstocks, trifoliate orange (TO), ‘Ziyang Xiangcheng’ (XC), red tangerine (RT), and ‘Shatian’ pummelo (ST), were grown under normal and excess of Cu (187.5 μM, 125×) conditions. After 25 d of treatment, representative pictures were photographed. Plant height increase rate and malonaldehyde (MDA) content were determined at 40 d, while chlorophyll a (chla) and b (chlb) contents were measured at 0 d, 25 d, and 40 d. Data are means ± SE (*n* = 3). Different letters indicate significant differences at *P* < 0.05 by Tukey test

To compare tolerance to Cu toxicity, four widely used citrus rootstocks, trifoliate orange (TO), ‘Ziyang Xiangcheng’ (XC), red tangerine (RT), and ‘Shatian’ pummelo (ST), were subjected to excessive Cu treatment, followed by evaluation of plant phenotypic and physiological parameters. As shown in Fig. 1, after 25 d of treatment, top leaves of TO and ST showed a yellow color, while those of XC and RT were almost normal (similar to the phenotype of CK). Although the relative increase rate of plant height was significantly suppressed upon Cu toxicity, XC had a minimal impact among the four rootstocks. Chlorophyll contents were also significantly reduced by Cu treatment, but XC had a higher level than the other three rootstocks at 25 and 40 d of treatment. In addition, excessive Cu treatment resulted in a drastic increase of MDA in TO, RT, and ST relative to CK, but increase of MDA in XC was not conspicuous. These results indicated that XC was the most tolerant genotype to Cu toxicity among the tested rootstocks. Therefore, XC was selected for high-throughput RNAseq to reveal global transcriptome of mRNA, lncRNA, circRNA and miRNA in response to Cu toxicity.

### Global responses of mRNA to Cu toxicity

**Fig. 2:**
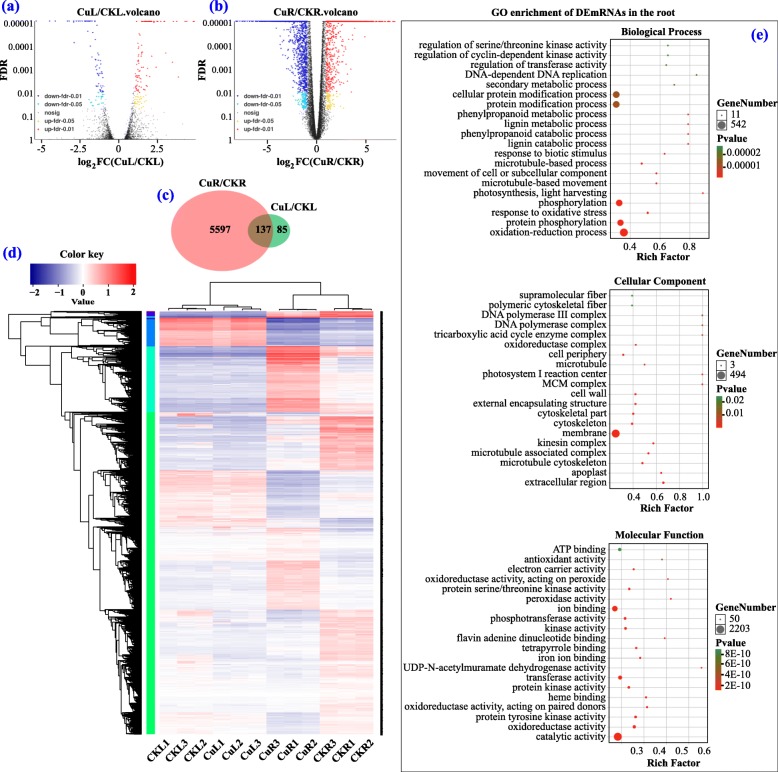
Identification and analysis of differentially expressed mRNAs (DEmRNAs) under Cu toxicity. **a**, **b** Volcano Plot pictures showing log_2_FC values and FDR of mRNAs in CuL/CKL and CuR/CKR. **c** Venn diagram showing the number of DEmRNAs in CuL/CKL and CuR/CKR. **d** Heat map of all DEmRNAs. **e** GO enrichment of DEmRNAs in the root

From RNAseq data, we identified 30,123 protein-coding genes in leaves and roots of XC by using pummelo as reference genome, and their log_2_FC values are presented as Volcano Plot pictures in Fig. 2a and b, which ranged from − 8.2 to 7.9 in roots and from − 5.3 to 4.8 in leaves. Among all of these genes, 5734 (2162 up-regulated, 3572 down-regulated) and 222 (132 up-regulated, 90 down-regulated) DEmRNAs were identified in Cu-treated root (CuR/CKR) and leaf (CuL/CKL) groups, respectively (Fig. 2c and Additional file 1: Table S1). The number of DEmRNAs in the root was significantly higher than those in the leaf, suggesting that the root had more predominant responses to Cu toxicity. Moreover, 137 DEmRNAs were common between CuR/CKR and CuL/CKL, implying that they might participate in the basic response process under Cu toxicity. A heat map of DEmRNAs showed the general expression profiles of DEmRNAs in each treatment and also showed that the three repeats of each treatment always clustered together while the Cu-treated group and the CK group were clustered separately (Fig. 2d).

To explore the functions of the DEmRNAs, GO annotation and GO enrichment analysis were performed. Base on GO annotation, we found that most DEmRNAs in the leaf and root were annotated to the metabolic process, single-organism process, localization process and response to stimulus under biological process (BP), to membrane, cell, organelle, and extracellular region under cellular component (CC), and to binding, catalytic activity, transporter activity, antioxidant activity, transcription factor activity and nutrient reservoir activity under molecular function (MF) (Additional file 10: Figure S1a and b). In particular, there were 317 DEmRNAs in response to stimulus, 596 in the membrane, 45 involved in antioxidant activity, 1 with metallochaperone activity, 32 with molecular transducer activity, 126 nucleic acid binding transcription factors, 18 with nutrient reservoir activity, 19 with receptor activity, and 188 with transporter activity in the root (Additional file 8: Table S8). GO enrichment analysis showed that the oxidation-reduction process, phosphorylation process, photosynthesis process, lignin catabolic process, phenylpropanoid catabolic process, membrane, dehydrogenase activity, peroxidase activity, protein kinase activity, iron ion binding, and heme binding were significantly enriched in the leaf and root (Fig. 2e and Additional file 10: Figure S1c), and these GO terms of DEmRNAs possible have primarily participated in mitigation of Cu toxicity.

### Global responses of lncRNA to Cu toxicity

**Fig. 3:**
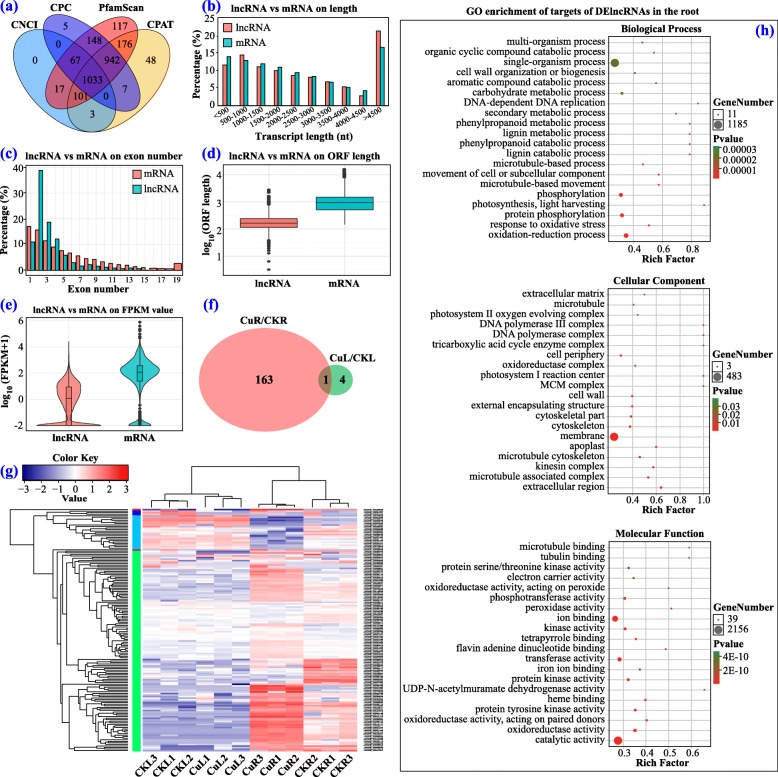
Identification and analysis of differentially expressed lncRNAs (DElncRNAs) under Cu toxicity. **a** Venn diagram showing the number of lncRNAs identified by CNCI, CPC, PfamScan and CPAT methods. **b**–**e** Comparison of lncRNA with mRNA with respect to the transcript length, exon number, ORF length and FPKM value. **f** Venn diagram showing the number of DElncRNAs in CuL/CKL and CuR/CKR. **g** Heat map of all DElncRNAs. **h** GO enrichment of targets of DElncRNAs in the root

Apart from mRNAs, we also identified 243 known lncRNAs and 1033 novel lncRNAs in XC from the RNAseq data by blasting to known lncRNAs of citrus in the GREENC database and performing CNCI, CPC, CPAT and PfamScan analysis (Fig. 3a). Comparison of the genomic characterizations of the lncRNAs with mRNAs showed that their transcripts were similar in length distribution, except lncRNA had relative higher numbers of long transcripts (> 4500 bp) than mRNA; for exon number, a higher percentage of lncRNAs had 2 to 4 exons; in addition, lncRNAs had a shorter ORF length and lower FPKM value (Fig. 3b-e). At a cutoff with an absolute value of log_2_FC > 1 and FDR < 0.05, 164 (103 up-regulated, 61 down-regulated) and 5 (1 up-regulated, 4 down-regulated) DElncRNAs were identified in CuR/CKR and CuL/CKL groups, respectively (Fig. 3f and Additional file 2: Table S2). The log_2_FC values of DElncRNAs ranged from − 10.0 to 13.2 in the root, and − 11.1 to 8.8 in the leaf. The general expression profiles of DElncRNAs are shown in Fig. 3g. Similar to DEmRNAs, the DElncRNAs in the Cu-treated group and CK group were clustered separately, while their three repeats were clustered together.

To explore the potential functions of these DElncRNAs, their *cis*- and *trans*-targeted mRNAs were predicted with bioinformatics methods (Additional file 3: Table S3). GO annotation of the targets of DElncRNAs in the root showed that they were annotated in 16 terms under BP (mainly involved in metabolic process, cellular process, single-organism process, localization and response to stimulus), 13 terms under CC (mainly involved in membrane, cell, organelle and macromolecular complex), and 10 terms under MF (mainly involved in binding, catalytic activity, transporter activity, electron carrier activity, transcription factor activity, and antioxidant activity) (Additional file 11: Figure S2). GO enrichment analysis of these targets showed that significantly enriched GO terms were the photosynthesis process, phosphorylation process, oxidation-reduction process, lignin catabolic process, phenylpropanoid catabolic process, MCM complex, photosystem I reaction center, extracellular region, membrane, dehydrogenase activity, peroxidase activity, protein kinase activity, iron ion binding, and heme binding (Fig. 3h).

### Global responses of circRNA to Cu toxicity

**Fig. 4:**
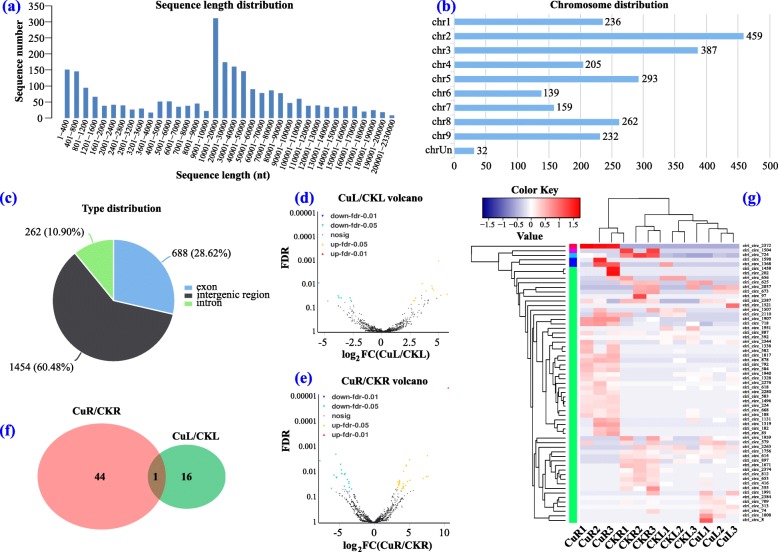
Identification and analysis of differentially expressed circRNAs (DEcircRNAs) under Cu toxicity. **a**–**c** Sequence length, chromosome, and type distribution of all identified circRNAs. **d**, **e** Volcano Plot pictures showing log_2_FC values and FDR of circRNAs in CuL/CKL and CuR/CKR. **f** Venn diagram showing the number of DEcircRNAs in CuL/CKL and CuR/CKR. **g** Heat map of all DEcircRNAs

In total, 2404 circRNAs were identified in the leaf and root of XC, and 60.48, 28.62, and 10.90% of them belong to the intergenic region type, exon type, and intron type, respectively (Fig. 4c). The sequence length distribution of circRNAs is shown in Fig. 4a, and most of them were 10,000 bp to 50,000 bp, or shorter than 1200 bp. Chromosome 2 (chr2) included maximum circRNAs, followed by chr3, chr5, and chr8 (Fig. 4b). Log_2_FC values of circRNAs are displayed in Fig. 4d and e, and 45 (28 up-regulated, 17 down-regulated) and 17 (11 up-regulated, 6 down-regulated) DEcircRNAs were identified in CuR/CKR and CuL/CKL groups, respectively (Fig. 4f and Additional file 4: Table S4), among which, 1 DEcircRNAs were common between CuR/CKR and CuL/CKL. A heat map showed the general expression profiles of DEcircRNAs in each treatment, and most DEcircRNAs were highly expressed in the CuR group (Fig. 4g). These results suggest that the circRNAs are also involved in the responses to Cu toxicity.

### Global responses of miRNA to Cu toxicity

**Fig. 5:**
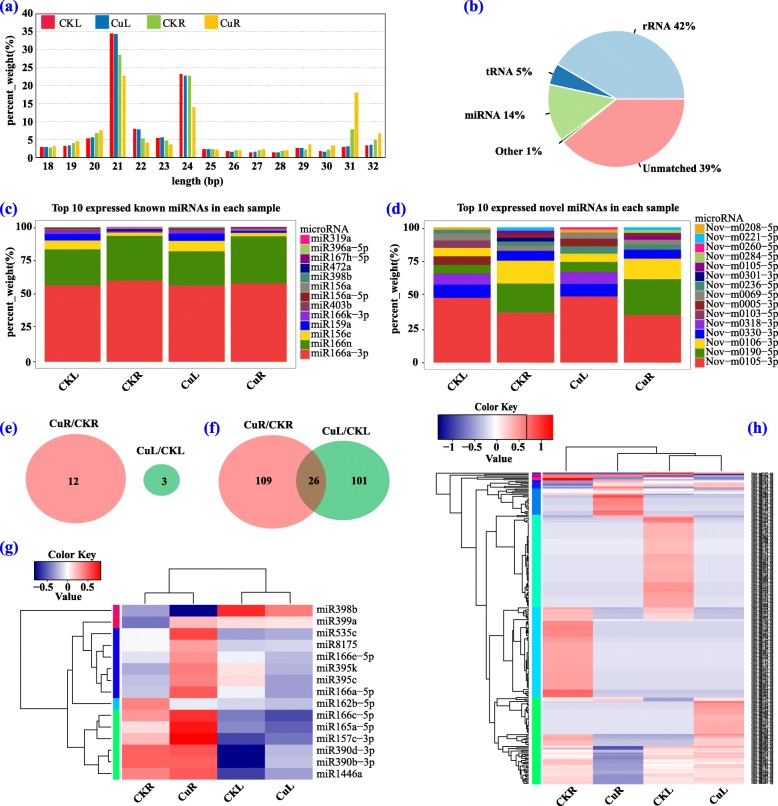
Identification and analysis of differentially expressed miRNAs (DEmiRNAs) under Cu toxicity. **a** Length distribution of all identified small RNAs. **b** Percentage of different types of small RNAs. **c**, **d** Top 10 expressed known and novel miRNAs in each sample. **e**, **f** Venn diagram showing the number of known (**e**) and novel (**f**) DEmiRNAs in CuL/CKL and CuR/CKR. **g**, **h** Heat map of all known (**g**) and novel (**h**) DEmiRNAs

In the present study, a total of 23,333,512, 24,526,156, 21,822,295, and 25,871,923 raw reads were generated from CKL, CKR, CuL and CuR small RNA libraries, respectively. Of these raw reads, we obtained 15,050,388, 15,173,260, 13,474,928 and 13,748,074 clean reads after removing adaptor sequences, low-quality sequences, and reads shorter than 18 nt and longer than 32 nt. The lengths of most clean reads were 20–24 nt (Fig. 5a). Small RNA classification showed that 81% of clean reads were rRNA (42%) and unmatched (39%), and there were also 14% miRNA, 5% tRNA, and 1% of other types (Fig. 5b). From 14% of clean reads, we finally identified 149 known miRNAs and 336 novel miRNAs. The top 10 expressed miRNAs in each sample are shown in Fig. 5c and d, and miR166a-3p and nov-m0105-3p exhibited the highest expression abundance. From known miRNAs 12 (10 up-regulated, 2 down-regulated) and 3 (all up-regulated) DEmiRNAs, and from novel miRNAs 135 (26 up-regulated, 109 down-regulated) and 127 (42 up-regulated, 85 down-regulated) DEmiRNAs were identified in CuR/CKR and CuL/CKL groups, respectively (Fig. 5e and f and Additional file 5: Table S5). The general expression profiles of these DEmiRNAs are shown in Fig. 5g and h. Their expression levels exhibited obvious differences between CK and Cu-treated samples and between root and leaf samples.

**Fig. 6:**
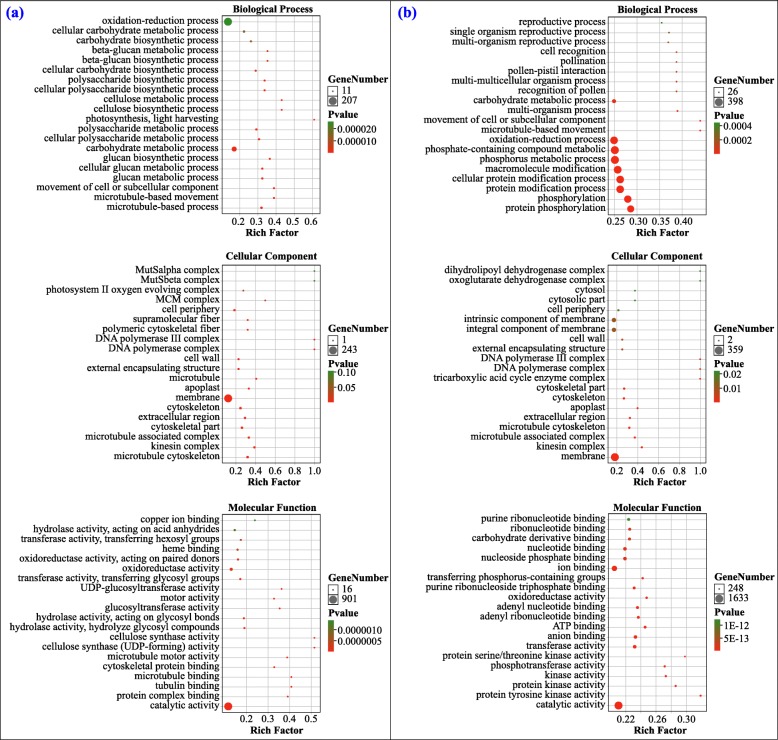
GO enrichment of targets of known (**a**) and novel (**b**) DEmiRNAs in the root

Targeted mRNAs of these DEmiRNAs are listed in Additional file 6: Table S6. We found that 84.7% of DEmRNAs in the leaf (188/222) and 81.0% of DEmRNAs in the root (4642/5734) were targeted by one or multiple DEmiRNAs. GO annotation of the targets of DEmiRNAs in the root showed that most of them were annotated to the metabolic process, cellular process, single-organism process, localization process, and response to stimulus under BP, to membrane, cell, organelle and macromolecular complex under CC, and to binding, catalytic activity and transporter activity under MF (Additional file 12: Figure S3a and b). GO enrichment analysis showed that the significantly enriched GO terms of the targets of known DEmiRNAs in root were photosynthesis, microtubule-based movement, carbohydrate metabolic process, DNA polymerase complex, microtubule, membrane, cellulose synthase activity, microtubule binding, and catalytic activity, while those of novel DEmiRNAs were microtubule-based movement process, phosphorylation process, protein modification process, oxidation-reduction process, DNA polymerase complex, kinesin complex, extracellular region, membrane, protein kinase activity, transferase activity, anion binding, and catalytic activity (Fig. 6a and b).

### CeRNA regulatory network in response to Cu toxicity

**Fig. 7:**
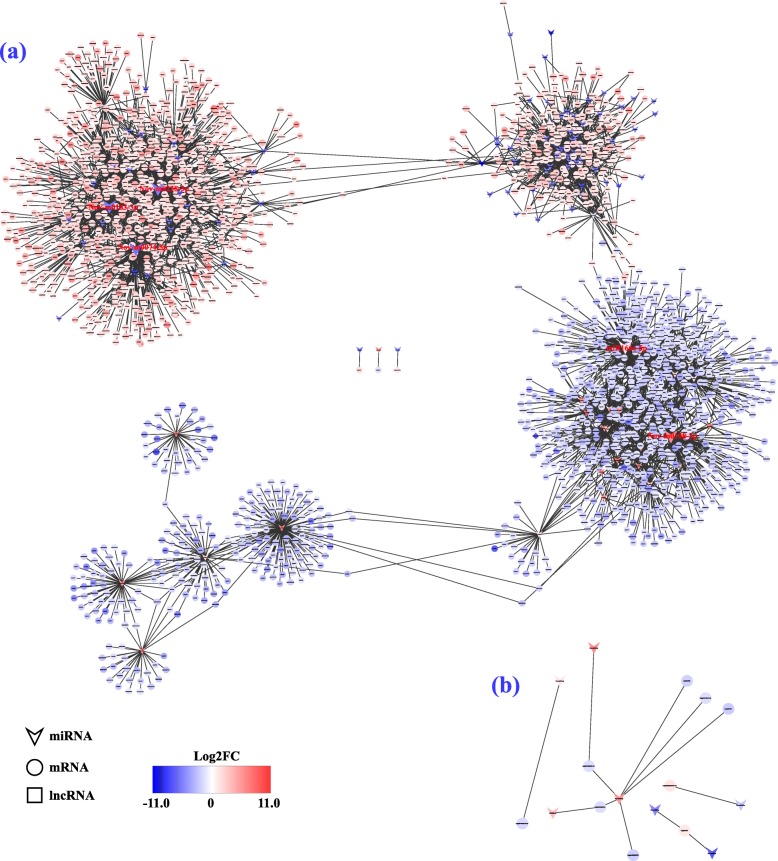
CeRNA network constructed with all DEmRNAs, DElncRNAs and DEmiRNAs in the root (**a**) and leaf (**b**). Color represents the up-regulated (red color) and down-regulated (blue color) levels

To reveal the global regulatory network of protein-coding RNAs and ncRNAs under Cu toxicity, a ceRNA network was constructed using DEmiRNAs, DEmRNAs, DElncRNAs, and DEcircRNAs based on ceRNA theory. In total, 5739 DEmRNAs, 64 DElncRNAs, and 5 DEcircRNAs were predicted as targets of 251 miRNAs in the root and leaf. When their correlation was further filtered with SCC < − 0.5, we obtained 3819 DEmiRNA-DEmRNA and 10 DEmiRNA-DElncRNA interactions in the root and 12 DEmiRNA-DEmRNA interactions in the leaf (Fig. 7). In this ceRNA network, Nov-m0238-3p, Nov-m0074-5p, Nov-m0183-3p, miR166c-5p, Nov-m0128-3p, Nov-m0328-5p, miR165a-5p, and miR535c are involved in more than 100 nodes, suggesting that they may act as core regulators. In addition, lncRNAs including TCONS_00012501, TCONS_00012960, TCONS_00025983, TCONS_00027274, TCONS_00034874, TCONS_00036810, TCONS_00042747, TCONS_00051884 and TCONS_00062474 participated in the network.

**Fig. 8:**
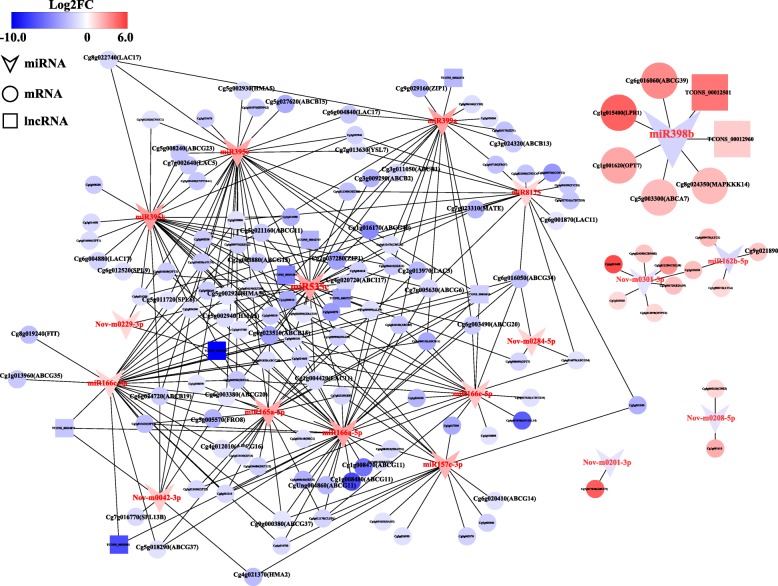
Mini-ceRNA network constructed with 284 known Cu-related DEmRNAs and all DElncRNAs and DEmiRNAs in the root. Color represents the up-regulated (red color) and down-regulated (blue color) levels

Considering that the network contains enormous information and each one cannot be displayed in the figure, we constructed a mini-ceRNA network by reducing the mRNA amount. We identified 284 known Cu-related mRNAs that were reported directly or indirectly in previous literatures from all DEmRNAs of the root; they included Cu-related regulators (SPL, YSL, CDPK, MAPK, and SUMO E3 Ligase, etc.), transporters (COPT, HMA, PPA, CDF, ZIP, OPT, and ABC transporter, etc.) and enzymes (LAC, CSD, CCS, PC, and COX, etc.) according to their functional description (Additional file 7: Table S7 and Additional file 8: Table S8). The mini-ceRNA network was constructed with these 284 DEmRNAs and all DEmiRNAs, DElncRNAs, and DEcircRNAs. Finally, only 261 DEmiRNA-DEmRNA and 10 DEmiRNA-DElncRNA interactions (SCC < − 0.5) containing 18 DEmiRNAs, 129 DEmRNAs, and 9 DElncRNAs were obtained and are displayed in Fig. 8. DEmiRNAs including miR166a-5p, miR395c, miR535c, miR395k, miR166c-5p, miR165a-5p, and miR399a were involved in more than 20 nodes, and all of them were up-regulated in the CuR. A known Cu-related key miRNA, miR398b, was identified in the network, which down-regulated and interacted with 5 DEmRNAs and 2 DElncRNAs. In addition, many known Cu-related key mRNAs such as SPL (Cg5g011720, Cg6g012520, Cg7g016770), YSL (Cg7g013630), HMA (Cg5g002920, Cg5g002930, Cg4g021370), ABC transporter (Cg5g018290, Cg5g027620, Cg3g011050, Cg3g009290, and Cg5g021160 etc.), LAC (Cg6g004840, Cg7g002640, Cg6g004880) and ZIP (Cg8g019240, Cg9g029160, Cg2g037280) were down-regulated in the network.

### qRT-PCR validated expression correlation between miRNAs and ceRNAs under Cu toxicity

To confirm the results of RNAseq and validate expression correlation of miRNA and their targets, six miRNAs (miR398b, miR8175, miR157c-3p, miR166a-5p, miR165a-5p, and Nov-m0284-5p) and their targeted mRNAs and lncRNAs were selected from the ceRNA network for qRT-PCR. As shown in Table 1, the qRT-PCR results agreed well with RNAseq data except several low-abundance mRNAs were undetectable. In addition, the miRNA and its targets showed quite correct up- or down-regulated relationships. For example, miR398 was down-regulated and all of its predicted targets were up-regulated; miR8175 was up-regulated and all of its targets were down-regulated. This result not only suggests reliability of RNAseq data in this study, but also validates the negatively correlated expression between miRNAs and ceRNAs.

**Table 1:** Confirmation of expression levels of miRNAs and their targeted lncRNAs and mRNAs based on qRT-PCR

	RNA	log_2_FC(CuR/CKR) by RNAseq	log_2_FC(CuR/CKR) by qPCR	*A. thaliana* description
	miR398b	-1.72561	-1.22962	
predicted targets of miR398b	TCONS_00012501	3.6	4.77311	
	TCONS_00012960	1.32	1.392306	
	Cg6g016060	3.06	3.721868	ABC transporter G family member 39
	Cg1g001620	2.35	2.21382	Oligopeptide transporter 7
	Cg1g015400	4.19	3.447331	Multicopper oxidase LPR1
	Cg5g003300	1.76	1.164213	ABC transporter A family member 7
	Cg8g024350	1.82	undetected	Mitogen-activated protein kinase kinase 14
	miR8175	1.089447	1.058019	
predicted targets of miR8175	Cg6g001870	-1.02	undetected	Laccase-11
	Cg4g001090	-2.13	-1.88539	Protein TIC 55, chloroplastic
	Cg9g027310	-2.25	-2.70294	Detoxifying efflux carrier 35
	Cg2g021840	-2.35	-4.28181	Cyclic nucleotide-gated ion channel 4
	Cg6g016050	-2.39	-1.89089	ABC transporter G family member 34
	Cg2g047100	-2.4	-2.92599	Ferric reduction oxidase 7
	Cg7g023310	-2.83	-2.96635	MATE efflux family protein 1
	Cg2g001540	-3.09	-4.80108	Transducin/WD40 repeat-like superfamily protein
	Cg6g005760	-4.11	-4.6826	Copper transporter 1
	miR157c-3p	1.98457	1.730227	
predicted targets of miR157c-3p	Cg5g033620	-1.1	-2.39108	E3 ubiquitin-protein ligase BAH1
	Cg6g020410	-1.21	-1.88539	ABC transporter G family member 14
	Cg5g026950	-1.23	undetected	RING/U-box superfamily protein
	Cg6g016780	-1.54	-2.09326	RCC1family with FYVE zinc finger domain
	Cg5g011170	-1.72	-1.56999	Chaperone protein ClpB1
	Cg7g023250	-1.76	-1.79581	E3 ubiquitin ligase BIG BROTHER
	Cg9g005380	-1.81	-1.71421	Copper amine oxidase family protein
	Cg9g005370	-1.83	-1.90385	Copper amine oxidase family protein
	Cg1g009950	-2.04	-3.01473	calmodulin-binding family protein
	Cg4g021370	-2.65	-3.17684	Cadmium/zinc-transporting ATPase HMA2
	Cg2g001540	-3.09	-4.80108	Transducin/WD40 repeat-like superfamily protein
	Cg2g017030	-3.4	-4.59911	Transducin/WD40 repeat-like superfamily protein
	Cg6g016070	-1.13	-1.13584	ABC transporter G family member 34
	Nov-m0284-5p	1.198039	1.01773	
predicted targets of Nov-m0284-5p	Cg6g016070	-1.13	-1.13584	ABC transporter G family member 34
	Cg5g000690	-1.7	undetected	Oligopeptide transporter 7
	Cg6g016050	-2.39	-1.89089	ABC transporter G family member 34
	miR166a-5p	2.37994	2.444839	
predicted targets of miR166a-5p	TCONS_00025983	-7.16	-3.31462	
	TCONS_00034874	-2.3	-2.30161	
	TCONS_00036810	-1.92	-1.70539	
	Cg2g034240	-1.17	-2.05887	MATE efflux family protein
	Cg3g013610	-2.61	-3.0194	Oligopeptide transporter 4
	Cg3g013620	-1.5	-1.66687	Oligopeptide transporter 2
	Cg5g001820	-1.35	undetected	ABC transporter C family member 10
	Cg5g008640	-3.08	-3.69684	Monocopper oxidase-like protein SKU5
	Cg5g011170	-1.72	-1.56999	Chaperone protein ClpB1
	Cg5g018290	-1.11	-0.16429	ABC transporter G family member 37
	Cg6g006410	-2.24	-3.65275	Laccase-4
	Cg6g016050	-2.39	-1.89089	ABC transporter G family member 34
	Cg6g016070	-1.13	-1.13584	ABC transporter G family member 34
	Cg7g005630	-1.6	-1.39821	ABC transporter G family member 6
	Cg7g023250	-1.76	-1.79581	E3 ubiquitin ligase BIG BROTHER
	Cg8g001210	-1.53	undetected	Major facilitator superfamily protein
	Cg9g000380	-1.08	undetected	ABC transporter G family member 37
	miR165a-5p	2.203897	2.322514	
predicted targets of miR165a-5p	TCONS_00036810	-1.92	-1.70539	
	Cg2g013970	-2.75	-2.55165	Laccase-3
	Cg3g013430	-1.32	-1.66301	MATE efflux family protein
	Cg3g013610	-2.61	-3.01937	Oligopeptide transporter 4
	Cg3g013620	-1.5	-1.66687	Oligopeptide transporter 2
	Cg3g024660	-1.87	-2.19929	Major facilitator superfamily protein
	Cg3g024680	-1.96	-2.62043	Plastocyanin major isoform, chloroplastic
	Cg5g011170	-1.72	-1.56999	Chaperone protein ClpB1
	Cg5g018290	-1.11	-0.16429	ABC transporter G family member 37
	Cg6g016050	-2.39	-1.89089	ABC transporter G family member 34
	Cg7g000950	-2.35	-2.44466	Laccase-4
	Cg7g023250	-1.76	-1.79581	E3 ubiquitin ligase BIG BROTHER
	Cg8g001210	-1.53	undetected	Major facilitator superfamily protein
	Cg8g024760	-1.34	-0.91518	Calcineurin-like metallo-phosphoesterase superfamily protein
	Cg9g000380	-1.08	undetected	ABC transporter G family member 37

### Expression patterns of candidate RNAs between XC and TO

**Fig. 9:**
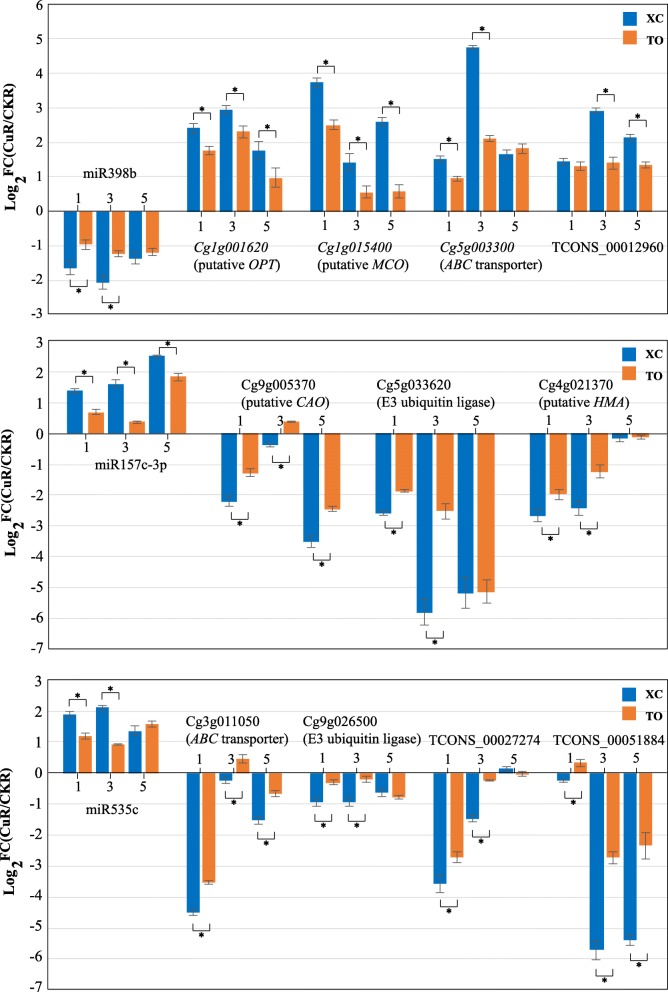
qRT-PCR analysis of the candidate miRNAs, mRNAs and lncRNAs in ‘Ziyang Xiangcheng’ (XC) and trifoliate orange (TO). Three miRNAs and their predicted targets were selected to determine the expression in roots of XC and TO which were treated with excess and normal Cu for 1 d, 3 d and 5 d. FC represents the fold changes of the relative expression levels between CuR and CKR. The data are the means ± SE of three technical replicates. Asterisk (*) indicates the significant difference at *P* < 0.05 by LSD testing

To further understand the Cu tolerance mechanism of XC, comparative analysis of the expression of known Cu-related miRNAs and ceRNAs was performed between Cu-tolerant XC and Cu-sensitive TO. As shown in Fig. 9, miR398b and its targets (Cg1g001620, Cg1g015400, Cg5g003300 and TCONS_0012960) were down-regulated or up-regulated in both roots of XC and TO at 1 d, 3 d and 5 d of Cu toxicity. However, the absolute values of log_2_FC in XC were significantly higher than those in TO at most time points. The similar changes were found for miR157c-3P and miR535c, as well as their targeted mRNAs and lncRNAs. These results suggest that the Cu-tolerant XC had occurred much more drastic expression of the Cu-related miRNAs, mRNAs and lncRNAs under Cu toxicity, which may be the important reason that leads to higher Cu tolerance in XC.

## Discussion

The ceRNA hypothesis is now widely accepted since it was reported several years ago [31]. Despite great progress in understanding human disease using the ceRNA theory [41], relatively fewer studies have been carried out in plants. In the ceRNA network, miRNAs play critical role in connection and regulation of different ceRNAs, such as mRNAs, lncRNAs and circRNAs. The mRNAs can be transcribed into proteins that exhibit direct functions, whereas the lncRNAs and circRNAs, not transcribed, can indirectly influence the expression of mRNAs by competitively binding the common miRNAs [31]. Based on this theory, we try to construct a ceRNA regulatory network so as to elucidate the mechanisms underlying tolerance to excessive Cu in the tolerant genotype.

In this study, 96.3% of DEmRNAs were predicted as the targets of 251 DEmiRNAs, which constitute 3819 DEmiRNA-DEmRNA interactions in the root and 12 in the leaf. This result suggests that most of the DEmRNAs may be regulated by the miRNAs under Cu toxicity. From the constructed ceRNA network, we found several miRNAs, including substantially down-regulated miR398b and up-regulated miR165a-5p, miR166a-5p, miR166c-5p, miR395c, miR395k, miR399a and miR535c, which have been previously reported to play a role in metal stress response. Among them, miR398 was shown to be significantly down-regulated under Cu excess but up-regulated under Cu deficiency [20, 22, 27, 42]. MiR395b and miR395c of *A. thaliana* were found to be up-regulated under Cu and cadmium (Cd) toxicity [43]. In addition, miR166 and miR399 were shown to play important roles in manganese (Mn), Cd, arsenic (As) or aluminum (Al) toxicity [44]. Although no direct evidence is provided to support the function of miR535 in metal stress, another member sharing same superfamily and high sequence similarity, miR156 has been well documented to function in Cd, Al, Mn, and As toxicity by targeting *SPL* genes [44, 45]. Interestingly, these metal stress-related miRNAs were predicted to target and regulate a large number of known metal stresses-related genes in the ceRNA network (Fig. 8, Additional file 7: Table S7 and Additional file 8: Table S8). For example, miR398b is predicted to target five DEmRNAs that significantly up-regulated by excessive Cu level, including putative ATP-binding cassette (ABC) transporters (Cg5g003300, Cg6g016060), oligopeptide transporter (OPT, Cg1g001620), multicopper oxidase (MCO, Cg1g015400), and mitogen-activated protein kinase kinase kinase (MAPKKK, Cg8g024350). It is well known that ABC transporters are one of the largest families of plant proteins that are suggested to be involved in metal ion uptake, transport and heavy metals detoxification [46, 47]. The OPT proteins consisting of YSL and PT clades have been demonstrated to participate in metal homeostasis through the translocation of metal-chelates [48]. The MCOs, which belong to the blue Cu proteins containing one to six Cu atoms per molecule, are suggested to function in redox reactions [49]. As an important signal transduction pathway, the MAPK signaling cascade plays key roles in response to different extracellular stimuli, and it has been previously found to be activated by excess of Cu and Cd [50, 51]. It is worth mentioning that except the miR398-targeted genes, other DEmRNAs, including putative SPLs, YSLs, HMAs, ABC transporters, LACs and ZIPs, were targeted by up-regulated miRNAs. These target mRNAs have also been shown to play essential roles in homeostasis of Cu or other metals. Therefore, we speculate that the better tolerance of Cu toxicity in XC is ascribed, at least in part, to miRNA-mediated regulation of the Cu-related genes, which allows it to maintain Cu homeostasis through alteration of signal transduction, Cu uptake, transport and detoxification, and antioxidant capacity. This speculation is corroborated by the differential expression levels of the miRNAs and targeted mRNAs in two genotypes with contrasting tolerance to Cu toxicity (Fig. 9).

Accumulating evidence indicates that the lncRNAs and circRNAs, two classes of new ncRNAs, act as ceRNAs (or named ‘miRNA sponges’, ‘target mimics’) to function in a variety of biological processes. In colorectal cancer, the lncRNA H19 was reported to derepress the endogenous genes, *Vimentin*, *ZEB1*, and *ZEB2*, by targeting miR138 and miR200a [52]. An autophagy promoting factor (APF) lncRNA was shown to interact with miR188-3p, and thus affected *ATG7* expression and autophagic cell death in heart [53]. In plants, overexpression of lncRNA IPS1 in *A. thaliana* led to increased accumulation of *PHO2*, which is targeted by miR399 and functions in phosphate uptake [54]. In addition, circRNAs have been revealed to function as miRNA sponges. For example, a circHIPK3 could sponge to nine miRNAs with 18 potential binding sites and significantly affected human cell growth [55]. In this study, we identified nine DElncRNAs in the ceRNA network, suggesting that these citrus lncRNAs may also function as miRNA sponges to participate in tolerance to Cu toxicity. However, it should be pointed out that most of DElncRNAs were not included in the ceRNA network. It is thus assumed that these lncRNAs involve in Cu toxicity via other mechanisms, such as transcribing as miRNA precursors, induction of DNA methylation, modulation of chromatin modification, or serving as transcription enhancers [56]. It is surpring and unexpected that all of the DEcircRNAs were not included in the ceRNA network. One of the possible reasons is that the criteria used to predict the ceRNA pairs and interactions between DERNAs is too stringent.

**Fig. 10:**
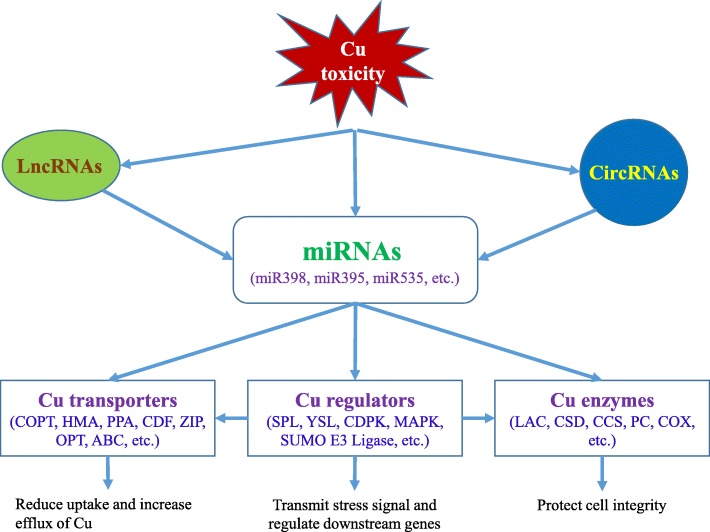
The proposed model in response to Cu toxicity in citrus. Under Cu toxicity, miRNAs act as the key regulators that directly target important Cu transporters, Cu proteins and Cu regulators (TFs and kinases). In this process, some lncRNAs and circRNAs can act as ceRNA to competitively bind the MRE of miRNAs, which may indirectly affect the expression of mRNAs

Based on the ceRNA theory and the ceRNA network constructed in this study, we propose a mode of action for the differentially expressed mRNAs, miRNAs, lncRNAs and circRNAs in response to the Cu toxicity (Fig. 10). Under Cu toxicity, miRNAs act as the key regulators to modulate up-regulation or down-regulation of important Cu transporters, Cu proteins and Cu regulators (TFs and kinases). As a consequence, uptake, efflux and distribution of Cu will be activated or suppressed, and cell integrity was protected, leading to enhanced tolerance to Cu toxicity. In this process, some lncRNAs can act as ceRNA to competitively bind the MRE of miRNAs, which may indirectly affect the expression of mRNA. Moreover, circRNAs possibly act as another type of ceRNA to sequester Cu-responsive miRNAs and suppress their function.

## Conclusions

Tolerance evaluation showed that XC was most tolerant to Cu toxicity. Whole-transcriptome RNAseq helped us to identify 5734 (2162 up-regulated, 3572 down-regulated) and 222 (132 up-regulated, 90 down-regulated) DEmRNAs in Cu-treated root and leaf, respectively, in which 1243 were considered to be key candidates in response to excessive Cu. We also identified 243 known and 1033 novel lncRNAs, of which 164 (103 up-regulated, 61 down-regulated) and 5 (1 up-regulated, 4 down-regulated) were significantly differentially expressed in the Cu-treated root and leaf, respectively. From 2404 identified circRNAs, only 45 (28 up-regulated, 17 down-regulated) and 17 (11 up-regulated, 6 down-regulated) DEcircRNAs were identified in root and leaf, respectively, exposed to excessive Cu. In addition, 149 known miRNAs and 336 novel miRNAs were predicted, and 147 and 130 of them were responsive to Cu toxicity in the root and leaf, respectively. GO enrichment analysis showed that most of the DEmRNAs and targets of DElncRNAs and DEmiRNAs were implicated in oxidation-reduction, phosphorylation, membrane, and ion binding. A ceRNA network consisting of differentially expressed mRNAs, miRNAs, and lncRNAs was constructed, which further revealed the critical roles of these DERNAs in tolerance to Cu toxicity.

## Methods

### Plant materials and treatments

Seeds of four commonly used citrus rootstocks, trifoliate orange (*Poncirus trifoliata* L. Raf.), ‘Ziyang Xiangcheng’ *(Citrus junos* Sieb. ex Tanaka), red tangerine (*C. reticulata* Blanco), and ‘Shatian’ pummelo (*C. grandis*) were collected from Citrus Research Institute of Southwest University (Chongqing, China). To evaluate the tolerance to Cu toxicity, seeds of the rootstocks were germinated in plastic containers filled with quartz sand at a temperature of 28 °C and a relative humidity of 80%. Uniform seedlings were transplanted into fresh quartz sand washed with distilled water at 25 °C under a 16-h photoperiod (50 μmol·m^− 2^·s^− 1^) for sand culture. During sand culture, ½-strength Hoagland’s solution composed of 4 mM Ca (NO_3_)_2_, 1.5 mM KNO_3_, 0.5 mM NH_4_H_2_PO_4_, 1 mM MgSO_4_, 50 μM Fe-EDTA, 15 μM H_3_BO_3_, 10 μM MnSO_4_, 5 μM ZnSO_4_, 1.5 μM CuSO_4_, and 1 μM H_2_MoO_4_, was irrigated every 4 d. After 30 d of growth, half of the seedlings were irrigated with the above mentioned solution (used as control, CK), while the rest seedlings were irrigated with ½-strength Hoagland’s solution containing 187.5 μM CuSO_4_ (125×) for 40 d, which was considered as Cu treatment. Three biological replicates were performed for each treatment.

For RNAseq and quantitative real time PCR (qRT-PCR) validation, seeds of ‘Ziyang Xiangcheng’ and trifoliate orange were first sterilized with 2% sodium hypochlorite for 15 min. After removal of seed coats, the seeds were germinated at a temperature of 28 °C and a relative humidity of 80% for one week. Uniform seedlings were transferred to the above-mentioned normal ½-strength Hoagland’s solution for hydroculture at 25 °C under a 16-h photoperiod (50 μmol·m^− 2^·s^− 1^), and the solution was renewed every 10 d. After 30 d of growth, half of the seedlings were renewed with ½-strength Hoagland’s solution containing 75 μM CuSO_4_ (50×) for excess Cu treatment, while the others were renewed with normal solution (CK). After 1 d, 3 d and 5 d of treatment, the roots and leaves of the seedlings were sampled, frozen in liquid nitrogen, and stored at − 80 °C, respectively. Three biological replicates were performed for each treatment.

### Measurement of plant height and contents of chlorophyll and malonaldehyde

Plant height (PH) of the aerial part was measured with a ruler at 0 (PH1) and 40 d of Cu treatment (PH2). Relative increase rate of the plant height was calculated as (PH2-PH1)/PH1 × 100%. Contents of chlorophyll and malonaldehyde (MDA) were determined as described by Fu et al. [57].

### RNA extraction, library preparation, and RNA sequencing

Total RNA was extracted from the roots and leaves of CK (abbreviated as CKR and CKL) and Cu-treated samples (abbreviated as CuR and CuL) using TRIzol Reagent (Invitrogen, Carlsbad, CA, USA) according to the manufacturer’s protocol. RNA quality and integrity were estimated with an Agilent 2100 Bioanalyzer (Agilent Technologies, Santa Clara, CA, USA) and NanoDrop 2000 spectrophotometer (Thermo Scientific, Wilmington, DE, USA). Only high-quality RNA samples (1.8 < OD260/280 < 2.2, OD260/230 ≥ 2.0, RIN ≥ 7.0, 28S/18S ≥ 1.0) were used to construct the sequencing library.

For mRNA, LncRNA, and circRNA sequencing, 5 μg of total RNA was used to prepare ribosomal RNA (rRNA) removed strand-specific library using a TruSeq Stranded Total RNA Library Prep with the Ribo-Zero Plant Kit (Illumina, San Diego, CA, USA) according to the manufacturer’s instructions. There were three biological replicates per treatment, and a total of 12 libraries were prepared. For small RNA sequencing, 4 small RNA libraries were constructed with 3 μg of total RNA from CKL, CKR, CuL and CuR samples and the Truseq Small RNA sample prep Kit (Illumina, San Diego, CA, USA). After libraries were quantified by a TBS-380 Fluorometer (Turner Biosystem, Sunnyvale, CA, USA), deep RNA sequencing was performed using an Illumina HiSeq X Ten platform at Shanghai Majorbio Bio-Pharm Biotechnology Co. Ltd. (Shanghai, China).

### Read mapping and transcriptome assembly

The paired-end raw reads were trimmed and quality controlled by SeqPrep (version 1.1, https://github.com/jstjohn/SeqPrep) and Sickle (version 1.33, https://github.com/najoshi/sickle) with default parameters. Then, clean reads were separately aligned to the Pummelo (*Citrus grandis*) genome [39] in orientation mode using Tophat2 software [58] (version 2.0.13, http://tophat.cbcb.umd.edu/). The mapped reads of each sample were assembled by Cufflink (version 2.2.1, http://cufflinks.cbcb.umd.edu/) in a reference-based approach.

### Differentially expressed mRNA and gene ontology (GO) enrichment analysis

To identify differentially expressed mRNAs (DEmRNAs) between CK and Cu-treated samples, the expression level of each transcript was calculated according to the fragments per kilobase of exon per million mapped reads (FRKM) method. RSEM (version 1.2.31, http://deweylab.biostat.wisc.edu/rsem/) was used to quantify gene abundance [59]. R statistical package software EdgeR (version 3.14.0) [60] was utilized for differential expression analysis with an absolute value of log_2_FC > 1 and FDR < 0.05. GO annotation and functional enrichment analysis of DEmRNAs were carried out using the Omicshare oneline platform (http://www.omicshare.com/tools/).

### Identification of lncRNAs and prediction of their target genes

For identification of novel lncRNAs, the transcripts that overlapped with known protein-coding genes on the same strand, transcripts with a fragment count < 3, transcripts shorter than 200 nt, and an open reading frame (ORF) longer than 300 nt were first discarded [61, 62]. Then, we used the Coding Potential Calculator (CPC, version 0.9), Coding-Non-Coding index (CNCI, version 2.0), Coding Potential Accessment Tool (CPAT, version 1.2.4), and Pfam Scan (version 1.6) to filter transcripts with coding potential. The overlapped outputs from CPC, CNCI, CPAT, and Pfam Scan were considered reliably expressed novel lncRNAs. The transcripts were also used to blast the citrus lncRNA sequences that were collected in the GREENC database (http://greenc.sciencedesigners.com/wiki/Main_Page), and the hits with e_value <1E-5 and matched bases ratio > 90% were identified as know lncRNAs. All identified lncRNAs were classified into intergenic, intronic, and antisense lncRNAs using the cuffcompare program in the Cufflinks suite. The expression level of each lncRNA was calculated according to the FRKM method. Differentially expressed lncRNAs (DELncRNAs) were extracted with an absolute value of log_2_FC > 1 and FDR < 0.05 by EdgeR. The potential *cis*- and *trans*-target mRNAs of DELncRNAs were predicted according to the position on the chromosome. The cis-targets were searched within a 10-kb window upstream or downstream of the lncRNA [63]. The *trans*-targets were predicted as described by Ou et al. [64].

### Identification and analysis of circRNAs

CircRNA Identifier (CIRI, version 1.2) and CIRCexplorer (version 1.1.7) tools were used to identify circRNAs in this study. CIRI scans Sequence Alignment/Map (SAM) files and detects junction reads with paired chiastic clipping (PCC) signals and paired-end mapping (PEM) and GT-AG splicing signals as described by Gao et al. [65]. CIRCexplorer obtains junction reads via a two-step TopHat and TopHat-Fusion mapping strategy as described by Zhang et al. [66]. The overlapped outputs from CIRI and CIRCexplorer were used for further analysis. The expression level of each circRNA was calculated according to the Spliced Reads per Billion Mapping (SRPBM) method. Differentially expressed circRNAs (DEcircRNAs) were extracted with an absolute value of log_2_FC > 1 and FDR < 0.05 by DEGseq (version 1.30.0).

### Identification and analysis of miRNAs

The raw data were first quality controlled with Fastx-toolkit software (version. 0.0.13, http://hannonlab.cshl.edu/fastx_toolkit/) to obtain clean small RNA reads by filtering low-quality bases (sanger base quality < 20), sequencing adapters, reads shorter than 18 nt, and reads longer than 32 nt. The assembled unique sequences with clean reads were then BLAST searched against the Rfam database (version 12.1, http://rfam.sanger.ac.uk/) to remove non-miRNA sequences (rRNA, tRNA, snoRNA, etc.). The remaining reads were used to predict known miRNAs through a BLAST search of the miRbase, version 21.0 (http://www.mirbase.org/), and novel miRNAs through analysis of the hairpin structure of the miRNA precursor with Mireap (version 0.2, http://sourceforge.net/projects/mireap) software. The expression level of each miRNA was calculated according to the transcripts per million reads (TPM) method. Differentially expressed miRNAs (DEmiRNAs) were extracted with an absolute value of log_2_FC > 1 and FDR < 0.005 by DEGseq. Target prediction of DEmiRNAs was performed with psRobot local version 1.01 [67].

### Construction and analysis of ceRNAs regulatory network

To reveal the roles and interactions of lncRNAs, circRNAs, miRNAs, and mRNAs, we constructed a lncRNA-circRNA-miRNA-mRNA regulatory network based on the ceRNA hypothesis. psRobot [67] was used to predict the pairs of miRNA-lncRNA, miRNA-mRNA, and miRNA-circRNA. The pairwise correlations of miRNA-lncRNA, miRNA-mRNA and miRNA-circRNA were evaluated using the Spearman correlation coefficient (SCC) and the matched pairs’ expression profile data as described by He et al. [40]. The interaction network was built with RNA pairs of SCC < − 0.5 and visually displayed using Cytoscape software (version 2.8) [68].

### qRT-PCR validation of DEmRNAs, DElncRNAs, and DEmiRNAs

qRT-PCR was performed to validate the expression levels of DEmRNAs, DElncRNAs, and DEmiRNAs. Total RNA and small RNA were extracted from samples of CuR and CKR using an RNAprep pure Plant Kit (TIANGEN, Cat#DP432, China) and miRcute miRNA isolation kit (TIANGEN, Cat#DP501, China), respectively. First-strand cDNA was synthesized from 1 μg of total RNA with the HiScript® II Q RT SuperMix (Vazyme, Cat#R223, China) for qPCR of mRNA and with the lnRcute lncRNA First-Strand cDNA Synthesis Kit (TIANGEN, Cat#KR202, China) for qPCR of lncRNA. In addition, 1 μg of small RNA was used for cDNA synthesis using a miRNA 1st Strand cDNA Synthesis Kit (Vazyme, Cat#MR101, China) with the stem-loop primer designed by the stem-loop sequence (GTCGTATCCAGGGTCCGAGGTATTCGCACTGGATACGAC) except for the internal reference U6. qPCR was performed on the Bio-Rad CFX Connect RealTime system using ChamQ™ Universal SYBR® qPCR Master Mix (Vazyme, Cat#Q711), lnRcute lncRNA qPCR Detection Kit (TIANGEN, Cat#FP402) and miRNA Universal SYBR® qPCR Master Mix (Vazyme, Cat#MQ101) following the manufacturer’s instructions. The 2^-ΔΔCT^ method was used to normalize and determine the RNA level relative to an internal reference gene, actin (Cs1g05000.1) or U6. All primers are included in Additional file 9: Table S9.

## Supplementary information

**Additional file 1: Table S1.** List of 5734 and 222 DEmRNAs in the root and leaf, respectively.

**Additional file 2: Table S2.** List of 164 and 5 DElncRNAs in the root and leaf, respectively.

**Additional file 3: Table S3.** *Cis* and *trans* targets of DElncRNAs.

**Additional file 4: Table S4.** List of 45 and 17 DEcircRNAs in the root and leaf, respectively.

**Additional file 5: Table S5.** List of 147 and 130 DEmiRNAs in the root and leaf, respectively.

**Additional file 6: Table S6.** DEmiRNAs targeted DEmRNAs in the root and leaf, respectively.

**Additional file 7: Table S7.** Searched known Cu-related DEmRNAs by using the keywords reported in previous studies to query their functional descriptions.

**Additional file 8: Table S8.** List of 1273 key DEmRNAs in response to Cu toxicity in citrus.

**Additional file 9: Table S9.** List of primers used in qRT-PCR.

**Additional file 10: Figure S1.** GO annotation of DEmRNAs in the leaf (a) and root (b), and GO enrichment of DEmRNAs in the leaf (c).

**Additional file 11: Figure S2.** GO annotation of targets of DElncRNAs in the root.

**Additional file 12: Figure S3.** GO annotation of targets of known (a) and novel (b) DEmiRNAs in the root.

## Data Availability

All supporting data can be found within the manuscript and its additional supporting files.

## Abbreviations

ABC transporter: ATP-binding cassette transporter
BP: Biological process
CC: Cellular component
ceRNAs: competing endogenous RNAs
circRNAs: circular RNAs
CIRI: circRNA identifier
CKL: Cu-untreated leaf
CKR: Cu-untreated root
CNCI: Coding-non-coding index
COPTs: CTR-like Cu transporters
COX: Cytochrome c oxidase
CPAT: Coding potential accessment tool
CPC: Coding potential calculator
CSD: Copper/zinc superoxide dismutases
Cu: Copper
CuL: Cu-treated leaf
CuR: Cu-treated root
DAO: Diamine oxidases
DE: Differentially expressed
FC: Fold change
FRKM: The fragments per kilobase of exon per million mapped reads
GO: Gene ontology
HMAs: P-type heavy metal ATPases
LAC: Laccase
lncRNAs: Long non-coding RNAs
MAPKKK: Mitogen-activated protein kinase kinase kinase
MCO: Multicopper oxidase
MDA: Malonaldehyde
MF: Molecular function
miRNAs: microRNAs
MREs: miRNA response elements
OPT: Oligopeptide transporter
ORF: Open reading frame
PC: Plastocyanin
PCC: Paired chiastic clipping
PEM: Paired-end mapping
PH: Plant height
qRT-PCR: Quantitative real time PCR
RNAseq: RNA sequencing
SAM: Sequence alignment/map
SCC: Spearman correlation coefficient
SPL7: Squamosa promoter binding-like 7
SRPBM: The spliced reads per billion mapping
TFs: Transcription factors
TO: Trifoliate orange
TPM: Transcripts per million reads
XC: ‘Ziyang Xiangcheng’
YSL: Yellow stripe-like protein

## References

[1] Broadley M, Brown P, Cakmak I, Rengel Z, Zhao F. Function of nutrients: Micronutrients. p. 191–248. In: P. Marschner (eds.), Mineral Nutrition of Higher Plants, Elsevier, 2012.

[2] Burkhead JL, Reynolds KAG, Abdel-Ghany SE, Cohu CM, Pilon M (2009). Copper homeostasis. New Phytol.

[3] Yruela Guerrero I (2009). Copper in plants: acquisition, transport and interactions. Funct Plant Biol.

[4] Yan J, Chia JC, Sheng H, Jung HI, Zhang L, Huang R, Jiao C, Craft EJ, Fei Z, Zavodna TO (2017). Arabidopsis pollen fertility requires the transcription factors CITF1 and SPL7 that regulate copper delivery to anthers and jasmonic acid synthesis. Plant Cell.

[5] Cambrolle J, Garcia JL, Figueroa ME, Cantos M (2015). Evaluating wild grapevine tolerance to copper toxicity. Chemosphere..

[6] Leng X, Jia H, Sun X, Shangguan L, Mu Q, Wang B, Fang J (2015). Comparative transcriptome analysis of grapevine in response to copper stress. Sci Rep.

[7] Huang XY, Deng F, Yamaji N, Pinson SR, Fujii-Kashino M, Danku J, Douglas A, Guerinot ML, Salt DE, Ma JF (2016). A heavy metal P-type ATPase OsHMA4 prevents copper accumulation in rice grain. Nat Commun.

[8] Zhao FJ, Ma Y, Zhu YG, Tang Z, McGrath SP (2015). Soil contamination in China: current status and mitigation strategies. Environ Sci Technol.

[9] Clemens S (2001). Molecular mechanisms of plant metal tolerance and homeostasis. Planta..

[10] Clemens S, Palmgren MG, Kramer U (2002). A long way ahead: understanding and engineering plant metal accumulation. Trends Plant Sci.

[11] Aguirre G, Pilon M (2016). Copper delivery to chloroplast proteins and its regulation. Front Plant Sci.

[12] Sancenon V, Puig S, Mateu-Andres I, Dorcey E, Thiele DJ, Penarrubia L (2004). The Arabidopsis copper transporter COPT1 functions in root elongation and pollen development. J Biol Chem.

[13] Jung HI, Gayomba SR, Rutzke MA, Craft E, Kochian LV, Vatamaniuk OK (2012). COPT6 is a plasma membrane transporter that functions in copper homeostasis in *Arabidopsis* and is a novel target of *SQUAMOSA* promoter-binding protein-like 7. J Biol Chem.

[14] Andres-Colas N, Sancenon V, Rodriguez-Navarro S, Mayo S, Thiele DJ, Ecker JR, Puig S, Penarrubia L (2006). The Arabidopsis heavy metal P-type ATPase HMA5 interacts with metallochaperones and functions in copper detoxification of roots. Plant J.

[15] Shikanai T, Muller-Moule P, Munekage Y, Niyogi KK, Pilon M (2003). PAA1, a P-type ATPase of Arabidopsis, functions in copper transport in chloroplasts. Plant Cell.

[16] Abdel-Ghany SE, Muller-Moule P, Niyogi KK, Pilon M, Shikanai T (2005). Two P-type ATPases are required for copper delivery in *Arabidopsis thaliana* chloroplasts. Plant Cell.

[17] Zheng L, Yamaji N, Yokosho K, Ma JF (2012). YSL16 is a phloem-localized transporter of the copper-nicotianamine complex that is responsible for copper distribution in rice. Plant Cell.

[18] Yamasaki H, Hayashi M, Fukazawa M, Kobayashi Y, Shikanai T (2009). SQUAMOSA promoter binding protein-like 7 is a central regulator for copper homeostasis in Arabidopsis. Plant Cell.

[19] Araki R, Mermod M, Yamasaki H, Kamiya T, Fujiwara T, Shikanai T (2018). SPL7 locally regulates copper-homeostasis-related genes in Arabidopsis. J Plant Physiol.

[20] Yamasaki H, Abdel-Ghany SE, Cohu CM, Kobayashi Y, Shikanai T, Pilon M (2007). Regulation of copper homeostasis by micro-RNA in Arabidopsis. J Biol Chem.

[21] Bernal M, Casero D, Singh V, Wilson GT, Grande A, Yang H, Dodani SC, Pellegrini M, Huijser P, Connolly EL (2012). Transcriptome sequencing identifies SPL7-regulated copper acquisition genes FRO4/FRO5 and the copper dependence of iron homeostasis in Arabidopsis. Plant Cell.

[22] Pilon M (2017). The copper microRNAs. New Phytol.

[23] Wang J, Meng X, Dobrovolskaya OB, Orlov YL, Chen M (2017). Non-coding RNAs and their roles in stress response in plants. Genom Proteom Bioinf.

[24] Chien PS, Chiang CB, Wang Z, Chiou TJ (2017). MicroRNA-mediated signaling and regulation of nutrient transport and utilization. Curr Opin Plant Biol.

[25] Abdel-Ghany SE, Pilon M (2008). MicroRNA-mediated systemic down-regulation of copper protein expression in response to low copper availability in Arabidopsis. J Biol Chem.

[26] Lu S, Li Q, Wei H, Chang M, Tunlaya-Anukit S, Kim H, Liu J, Song J, Sun YH, Yuan L (2013). Ptr-miR397a is a negative regulator of laccase genes affecting lignin content in *Populus trichocarpa*. P Natl Acad Sci USA.

[27] Sunkar R, Kapoor A, Zhu JK (2006). Posttranscriptional induction of two cu/Zn superoxide dismutase genes in Arabidopsis is mediated by downregulation of miR398 and important for oxidative stress tolerance. Plant Cell.

[28] Chekanova JA (2015). Long non-coding RNAs and their functions in plants. Curr Opin Plant Biol.

[29] Ma K, Shi W, Xu M, Liu J, Zhang F (2018). Genome-wide identification and characterization of long non-coding RNA in wheat roots in response to Ca^2+^ channel blocker. Front Plant Sci.

[30] Li QF, Zhang YC, Chen YQ, Yu Y (2017). Circular RNAs roll into the regulatory network of plants. Biochem Bioph Res.

[31] Salmena L, Poliseno L, Tay Y, Kats L, Pandolfi PP (2011). A ceRNA hypothesis: the Rosetta stone of a hidden RNA language?. Cell..

[32] Xu XW, Zhou XH, Wang RR, Peng WL, An Y, Chen LL (2016). Functional analysis of long intergenic non-coding RNAs in phosphate-starved rice using competing endogenous RNA network. Sci Rep.

[33] Zhu M, Zhang M, Xing L, Li W, Jiang H, Wang L, Xu M (2017). Transcriptomic analysis of long non-coding RNAs and coding genes uncovers a complex regulatory network that is involved in maize seed development. Genes..

[34] Ren GJ, Fan XC, Liu TL, Wang SS, Zhao GH (2018). Genome-wide analysis of differentially expressed profiles of mRNAs, lncRNAs and circRNAs during *Cryptosporidium baileyi* infection. BMC Genomics.

[35] Meng X, Zhang P, Chen Q, Wang J, Chen M (2018). Identification and characterization of ncRNA-associated ceRNA networks in Arabidopsis leaf development. BMC Genomics.

[36] Yuan Y, Li J, Xiang W, Liu Y, Shu J, Gou M, Qing M (2018). Analyzing the interactions of mRNAs, miRNAs, lncRNAs and circRNAs to predict competing endogenous RNA networks in glioblastoma. J Neuro-Oncol.

[37] Yang Z, Yang C, Wang Z, Yang Z, Chen D, Wu Y (2019). LncRNA expression profile and ceRNA analysis in tomato during flowering. PLoS One.

[38] Wang Y, Wang Q, Gao L, Zhu B, Luo Y, Deng Z, Zuo J (2017). Integrative analysis of circRNAs acting as ceRNAs involved in ethylene pathway in tomato. Physiol Plantarum.

[39] Wang X, Xu Y, Zhang S, Cao L, Huang Y, Cheng J, Wu G, Tian S, Chen C, Liu Y (2017). Genomic analyses of primitive, wild and cultivated citrus provide insights into asexual reproduction. Nat Genet.

[40] He X, Guo S, Wang Y, Wang L, Shu S, Sun J, et al. Physiol Plantarum. 2019. 10.1111/ppl.12997.

[41] Su XQ, Xing JD, Wang ZZ, Chen L, Cui M, Jiang BH (2013). microRNAs and ceRNAs: RNA networks in pathogenesis of cancer. Chinese J Cancer Res.

[42] Leng X, Wang P, Zhao P, Wang M, Cui L, Shangguan L, Wang C (2017). Conservation of microRNA-mediated regulatory networks in response to copper stress in grapevine. Plant Growth Regul.

[43] Gielen H, Remans T, Vangronsveld J, Cuypers A (2016). Toxicity responses of cu and cd: the involvement of miRNAs and the transcription factor SPL7. BMC Plant Biol.

[44] Gupta OP, Sharma P, Gupta RK, Sharma I (2014). MicroRNA mediated regulation of metal toxicity in plants: present status and future perspectives. Plant Mol Biol.

[45] Ding Y, Chen Z, Zhu C (2011). Microarray-based analysis of cadmium-responsive microRNAs in rice (*Oryza sativa*). J Exp Bot.

[46] Wojas S, Hennig J, Plaza S, Geisler M, Siemianowski O, Sklodowska A, Ruszczynska A, Bulska E, Antosiewicz DM (2009). Ectopic expression of Arabidopsis ABC transporter MRP7 modifies cadmium root-to-shoot transport and accumulation. Environ Pollut.

[47] Kim DY, Bovet L, Maeshima M, Martinoia E, Lee Y (2007). The ABC transporter AtPDR8 is a cadmium extrusion pump conferring heavy metal resistance. Plant J.

[48] Lubkowitz M (2011). The oligopeptide transporters: a small gene family with a diverse group of substrates and functions?. Mol Plant.

[49] Hoegger PJ, Kilaru S, James TY, Thacker JR, Kues U (2006). Phylogenetic comparison and classification of laccase and related multicopper oxidase protein sequences. FEBS J.

[50] Jonak C, Nakagami H, Hirt H (2004). Heavy metal stress. Activation of distinct mitogen-activated protein kinase pathways by copper and cadmium. Plant Physiol.

[51] Yeh CM, Hsiao LJ, Huang HJ (2004). Cadmium activates a mitogen-activated protein kinase gene and MBP kinases in rice. Plant Cell Physiol.

[52] Liang WC, Fu WM, Wong CW, Wang Y, Wang WM, Hu GX, Zhang L, Xiao LJ, Wan DCC, Zhang JF (2015). The lncRNA H19 promotes epithelial to mesenchymal transition by functioning as miRNA sponges in colorectal cancer. Oncotarget..

[53] Wang K, Liu CY, Zhou LY, Wang JX, Wang M, Zhao B, Zhao WK, Xu SJ, Fan LH, Zhang XJ (2015). APF lncRNA regulates autophagy and myocardial infarction by targeting miR-188-3p. Nat Commun.

[54] Franco-Zorrilla JM, Valli A, Todesco M, Mateos I, Puga MI, Rubio-Somoza I, Leyva A, Weigel D, Garcia JA, Paz-Ares J (2007). Target mimicry provides a new mechanism for regulation of microRNA activity. Nat Genet.

[55] Zheng QP, Bao CY, Guo WJ, Li SY, Chen J, Chen B, Luo YT, Lyu DB, Li Y, Shi GH (2016). Circular RNA profiling reveals an abundant circHIPK3 that regulates cell growth by sponging multiple miRNAs. Nat Commun.

[56] Hou J, Lu D, Mason AS, Li B, Xiao M, An S, Fu D (2019). Non-coding RNAs and transposable elements in plant genomes: emergence, regulatory mechanisms and roles in plant development and stress responses. Planta..

[57] Fu XZ, Khan EU, Hu SS, Fan QJ, Liu JH (2011). Overexpression of the betaine aldehyde dehydrogenase gene from *Atriplex hortensis* enhances salt tolerance in the transgenic trifoliate orange (*Poncirus trifoliata* L. Raf.). Environ Exp Bot.

[58] Kim D, Pertea G, Trapnell C, Pimentel H, Kelley R, Salzberg SL (2013). TopHat2: accurate alignment of transcriptomes in the presence of insertions, deletions and gene fusions. Genome Biol.

[59] Li B, Dewey CN (2011). RSEM: accurate transcript quantification from RNA-Seq data with or without a reference genome. BMC Bioinformatics.

[60] Robinson MD, McCarthy DJ, Smyth GK (2010). EdgeR: a bioconductor package for differential expression analysis of digital gene expression data. Bioinformatics.

[61] Ding ZH, Wu CL, Tie WW, Yan Y, He GY, Hu W (2019). Strand-specific RNA-seq based identification and functional prediction of lncRNAs in response to melatonin and simulated drought stresses in cassava. Plant Physiol Bioch.

[62] Quinn JJ, Chang HY (2016). Unique features of long non-coding RNA biogenesis and function. Nat Rev Genet.

[63] Jia H, Osak M, Bogu GK, Stanton LW, Johnson R, Lipovich L (2010). Genome-wide computational identification and manual annotation of human long noncoding RNA genes. RNA..

[64] Ou L, Liu Z, Zhang Z, Wei G, Zhang Y, Kang L, Yang B, Yang S, Lv J, Liu Y (2017). Noncoding and coding transcriptome analysis reveals the regulation roles of long noncoding RNAs in fruit development of hot pepper (*Capsicum annuum L*.). Plant Growth Regul.

[65] Gao Y, Wang J, Zhao F (2015). CIRI: an efficient and unbiased algorithm for de novo circular RNA identification. Genome Biol.

[66] Zhang XO, Wang HB, Zhang Y, Lu X, Chen LL, Yang L (2014). Complementary sequence-mediated exon circularization. Cell..

[67] Wu HJ, Ma YK, Chen T, Wang M, Wang XJ (2012). PsRobot: a web-based plant small RNA meta-analysis toolbox. Nucleic Acids Res.

[68] Shannon P, Markiel A, Ozier O, Baliga NS, Wang JT, Ramage D, Amin N, Schwikowski B, Ideker T (2003). Cytoscape: a software environment for integrated models of biomolecular interaction networks. Genome Res.

