# Supplementary figures and images for: Whole-transcriptome RNA sequencing reveals the global molecular responses and ceRNA regulatory network of mRNAs, lncRNAs, miRNAs and circRNAs in response to copper toxicity in Ziyang Xiangcheng (Citrus junos Sieb. Ex Tanaka)
Source: BMC Plant Biol. 2019 Nov 21;19:509. doi: 10.1186/s12870-019-2087-1 (PMC6873749; doi:10.1186/s12870-019-2087-1)

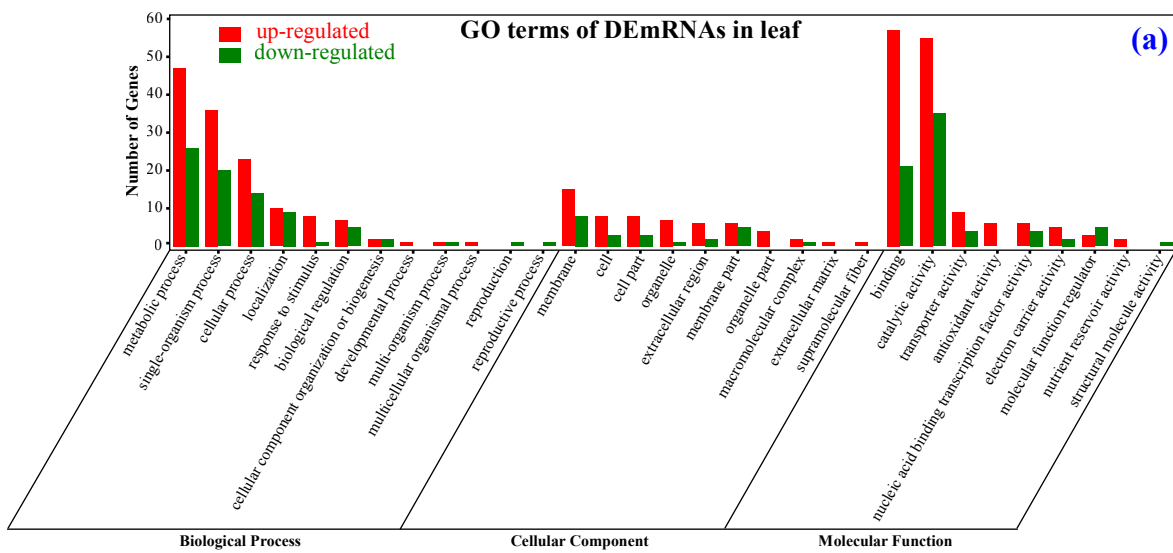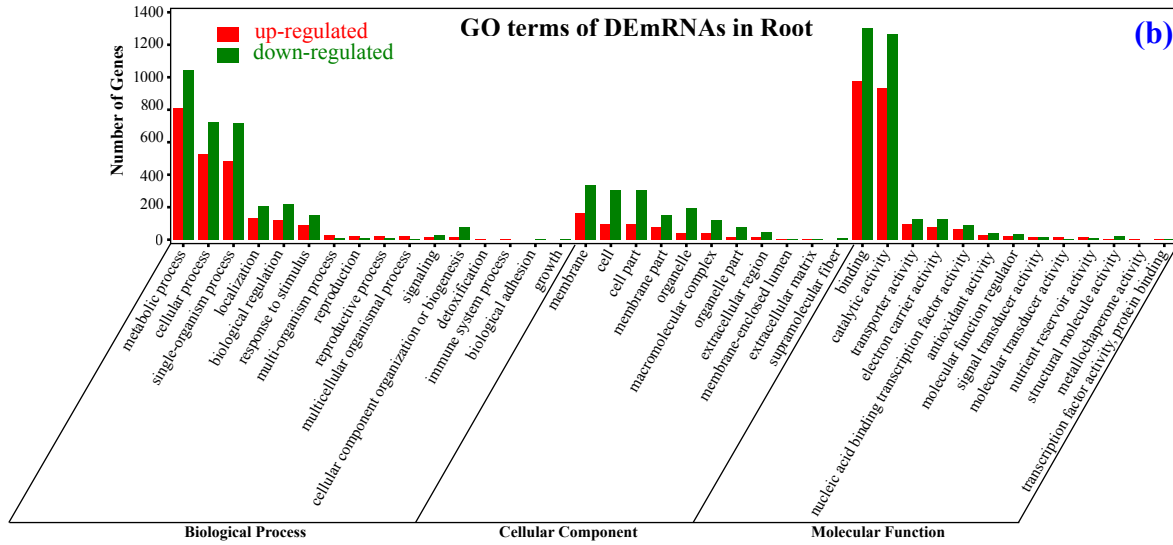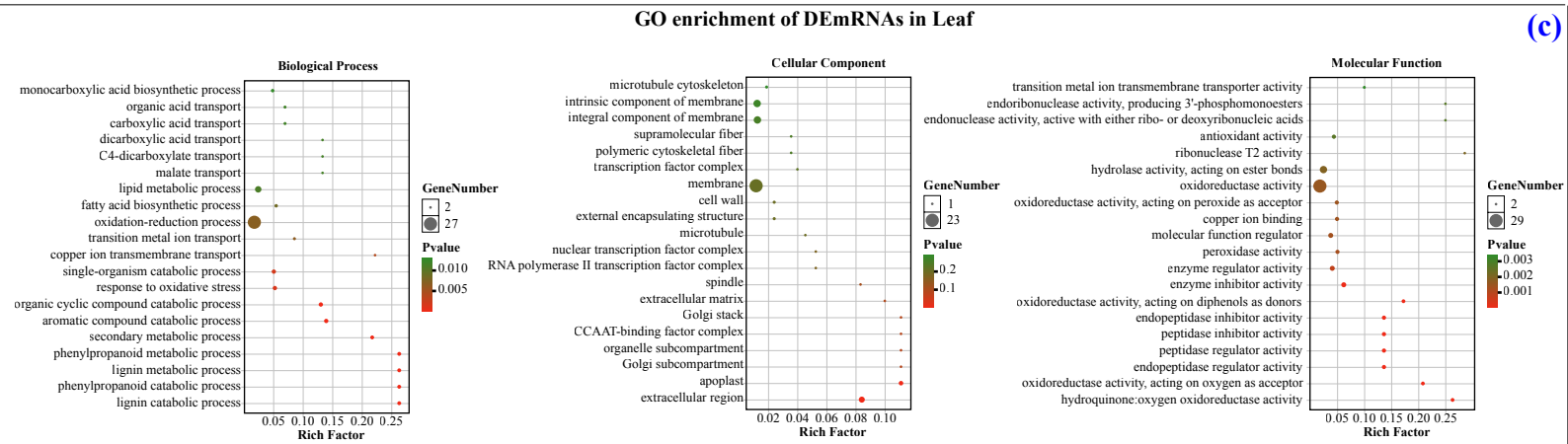

Supplement: Supplementary file 10 — Additional file 10: Figure S1. GO annotation of DEmRNAs in the leaf (a) and root (b), and GO enrichment of DEmRNAs in the leaf (c). [file 12870_2019_2087_MOESM10_ESM.pdf]

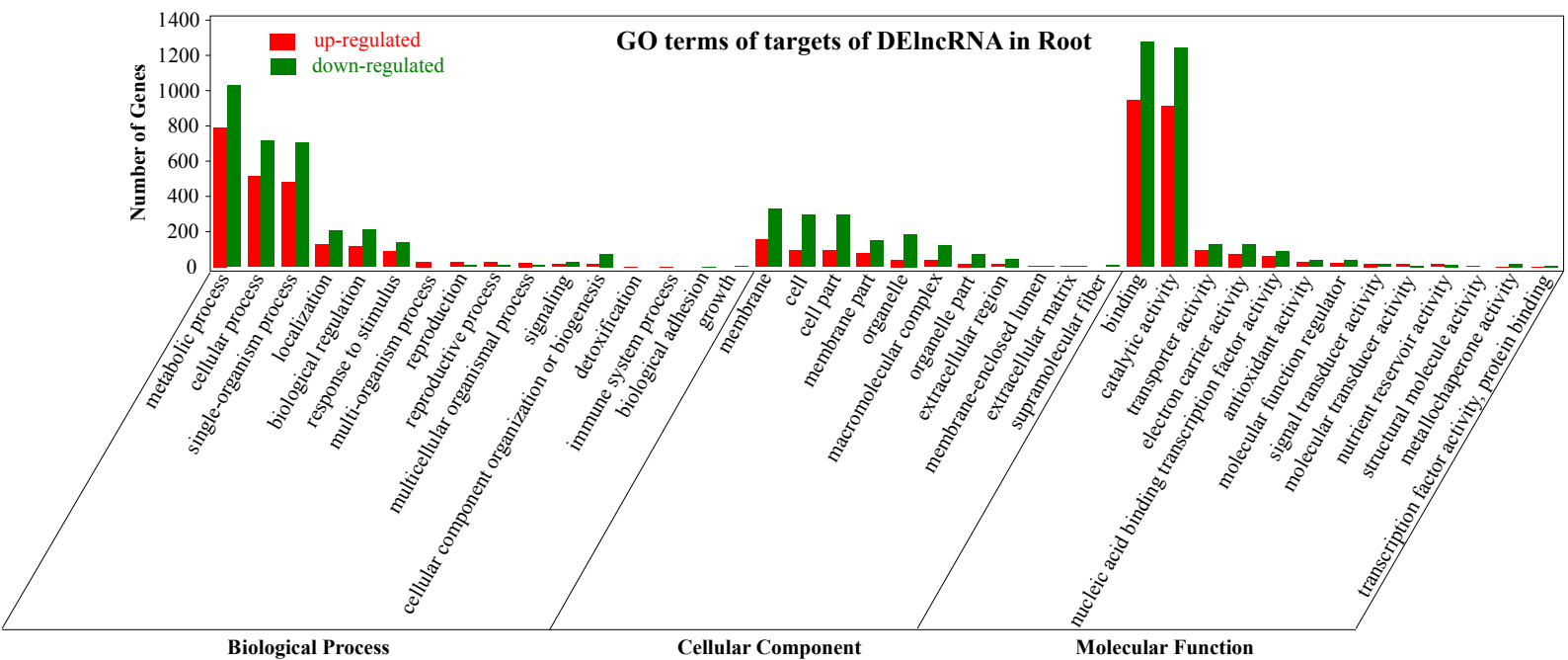

Supplement: Supplementary file 11 — Additional file 11: Figure S2. GO annotation of targets of DElncRNAs in the root. [file 12870_2019_2087_MOESM11_ESM.pdf]

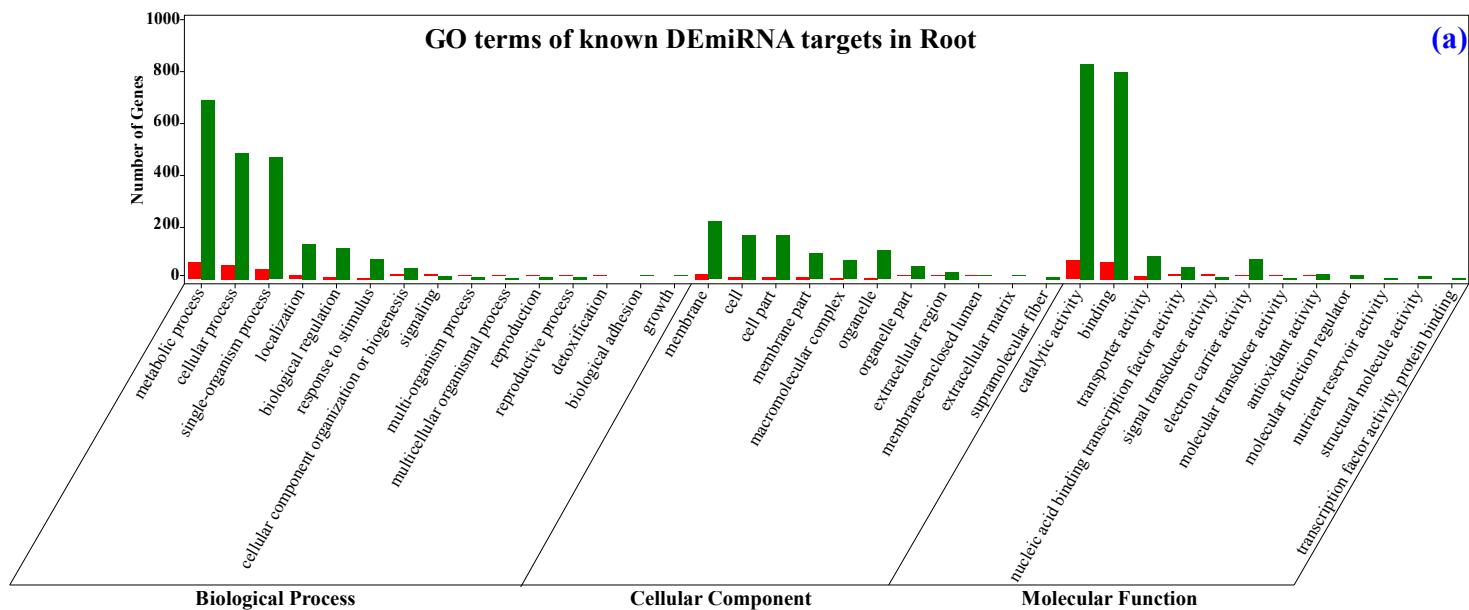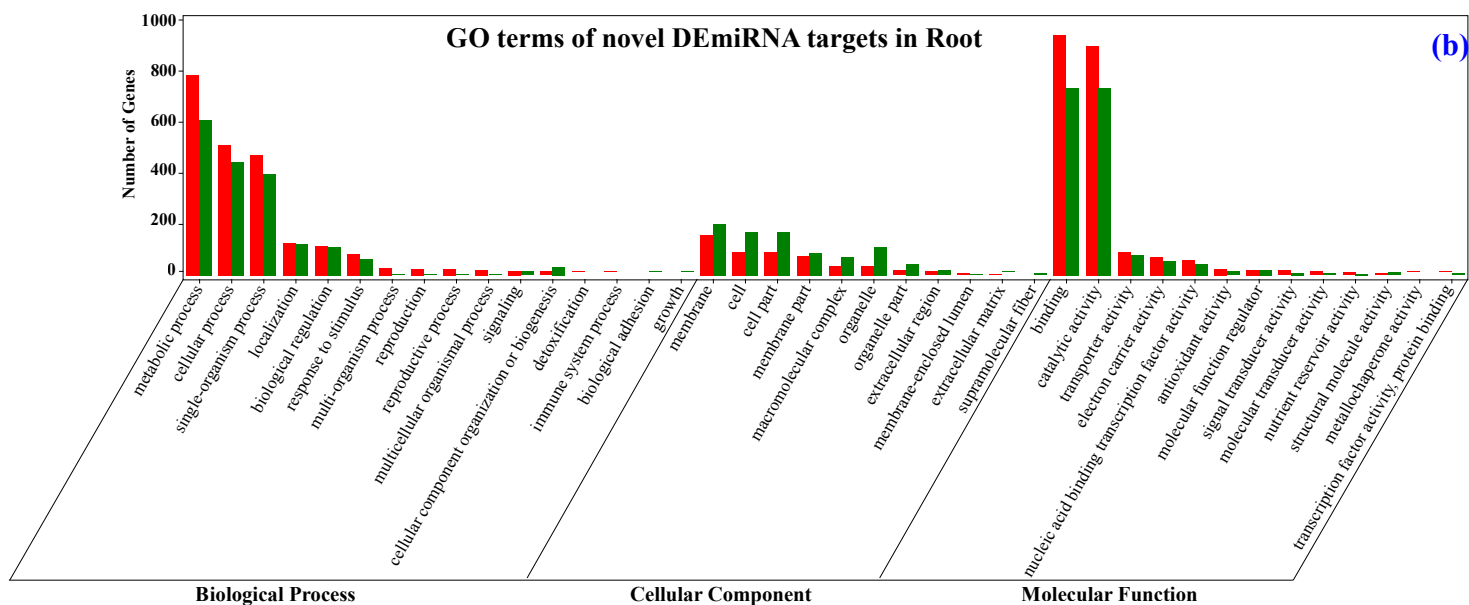

Supplement: Supplementary file 12 — Additional file 12: Figure S3. GO annotation of targets of known (a) and novel (b) DEmiRNAs in the root. [file 12870_2019_2087_MOESM12_ESM.pdf]
